# Is there a demand for physical activity interventions provided by the health care sector? Findings from a population survey

**DOI:** 10.1186/1471-2458-10-34

**Published:** 2010-01-25

**Authors:** Matti E Leijon, Diana Stark-Ekman, Per Nilsen, Kerstin Ekberg, Lars Walter, Agneta Ståhle, Preben Bendtsen

**Affiliations:** 1 Center for Primary Health Care Research, Lund University/Region Skåne, Malmö, Sweden; 2 Department of Medical and Health Sciences, Division of Community Medicine, Social Medicine and Public Health Science, Linköping University, Linköping, Sweden; 3 Department of Medical and Health Sciences, Division of Community Medicine, National Centre for Work and Rehabilitation, Linköping University, Linköping, Sweden; 4 Centre for Public Health Sciences, County Council of Östergötland, Linköping, Sweden; 5 Department of Neurobiology, Health Care Sciences and Society, Division of Physiotherapy, Karolinska Institutet, Stockholm, Sweden

## Abstract

**Background:**

Health care providers in many countries have delivered interventions to improve physical activity levels among their patients. Thus far, less is known about the population's interest to increase their physical activity levels and their opinion about the health care provider's role in physical activity promotion. The aims of this paper were to investigate the self-reported physical activity levels of the population and intention to increase physical activity levels, self-perceived need for support, and opinions about the responsibilities of both individuals and health care providers to promote physical activity.

**Methods:**

A regional public health survey was mailed to 13 440 adults (aged 18-84 years) living in Östergötland County (Sweden) in 2006. The survey was part of the regular effort by the regional Health Authorities.

**Results:**

About 25% of the population was categorised as physically active, 38% as moderately active, 27% as somewhat active, and 11% as low active. More than one-third (37%) had no intentions to increase their physical activity levels, 36% had thought about change, while 27% were determined to change. Lower intention to change was mainly associated with increased age and lower education levels. 28% answered that physical activity was the most important health-related behaviour to change "right now" and 15% of those answered that they wanted or needed support to make this change. Of respondents who might be assumed to be in greatest need of increased activity (i.e. respondents reporting poor general health, BMI>30, and inactivity) more than one-quarter wanted support to make improvements to their health. About half of the respondents who wanted support to increase their physical activity levels listed health care providers as a primary source for support.

**Conclusions:**

These findings suggest that there is considerable need for physical activity interventions in this population. Adults feel great responsibility for their own physical activity levels, but also attribute responsibility for promoting increased physical activity to health care practitioners.

## Background

Health-related behaviours such as physical activity, diet, alcohol use and smoking, contribute substantially to adult health status [1,2]. Numerous approaches have been taken at national and regional levels to influence adults to change their health-related behaviours in order to improve their health and quality of life. In recent years primary health care (PHC) providers have delivered interventions to improve physical activity levels among patients in many countries, including Sweden [3-9]. These interventions are often referred to as exercise prescriptions or physical activity referral (PAR) schemes [3,5,6,8]. Many of these interventions include brief face-to-face communication about the importance of physical activity along with written prescriptions for physical activity. These methods and interventions differ in content in different settings, but are generally regarded as acceptable and feasible, both to practitioners and patients [8,10]. Still, there is limited evidence, especially about the long term effect of these interventions [3,11,12]. In addition, little is known about which part of the population gains the most benefit from physical activity interventions [3] and about the patients' opinions about the role of health care providers in physical activity promotion.

This paper presents data from a regional public health survey conducted amongst adults living in Östergötland County in Sweden. The main purposes were to investigate the populations' self-reported 1) physical activity levels; 2) intentions to change their physical activity levels; 3) need for support; and 4) opinions on the responsibilities of both the individual and health care providers to promote physical activity in the adult population.

## Methods

A population survey was conducted in March 2006, as part of a regular effort by Health Authorities in Östergötland County, southern Sweden, to monitor health and the prevalence of risk factors in the general adult population. This county (population 416,000) is the fourth largest in Sweden with two large cities (>120,000 inhabitants combined) and 11 smaller, more rural municipalities. The survey's target population were county residents aged 18-84 years (n = 315 587). A questionnaire, consisting of 20 pages, was mailed to a sample of 13 440 individuals. Two reminders and an extra survey were sent to non-responders. The sample was stratified by gender, age, and municipalities resulting in 104 strata, with simple random sampling within each stratum [13]. The questionnaire and the survey were designed by the County Council's Public Health Department. The sample was administered by Statistics Sweden. A summary of the questions and the response items are presented in additional file 1. Ethical approval was not required, as data collection was part of the health care routine system.

The survey included a series of questions on health-related behaviour including questions about nutrition, physical activity, alcohol consumption, tobacco use and weight [14]. Physical activity was assessed by two questions (see additional file 1), one on physical activity in everyday life and one on exercise during the last 12 months. Responses from the two questions were categorised using a four-level physical activity index: low active, somewhat active, moderately active and physically active according to, or close to, public health recommendations level, normally set as 30 minutes of moderately intensive activity at least 5 days a week or 150 minutes a week or a more vigorously intensive activity at least 75 minutes a week [15-18]. The purpose of these measures is not primarily to provide precise estimates of the activity level, but more to differentiate between those who are more or less active.

Participants were asked if they had considered changing any health-related behaviour, which health risk behaviour they considered to be the most important to change "right now", and if they wanted or needed support to make these changes. If respondents wanted or needed support, they were asked to identify which support systems or care providers that would be most helpful in effecting change, including PHC, hospital, occupational health service, dentist, pharmacy, Internet, and a free text fill-in response alternative. Respondents were also asked questions regarding their opinions about personal responsibility for conducting a physically active lifestyle, and their opinions concerning health care providers' responsibilities in promoting physically active lifestyles (see additional file 1).

We combined many survey responses into larger categories. For example, responses measuring "support sources for change" were recombined into a dichotomous variable, consisting of "Health care" and "Others". The definition of "Health care" consisted of primary health care, hospital, occupational health service, and pharmacy; the "Others" category consisted of two responses: Internet and free text options not related to the health care service. Items asking respondents to agree with statements regarding responsibility for promoting physical activity in the adult populations included the following response alternatives: very much, somewhat, not much, very little. These alternatives are presented as a dichotomous variable; "Great" (including those who responded very much and somewhat) and "Little" (including those responding not much and very little). The self-reported economy question (a proxy for income or socio-economic position) included five response alternatives including "Neither good nor poor". The options "Very good" and "Rather good" are presented as "Good". "Very poor" and "Rather poor" are presented as "Poor". To measure general health status, we used one fill-in-the-blank question: "In general, would you say your health is?" Responses were dichotomised as: "Good" (comprised of those responding with "Excellent", "Very good" and "good") or "Poor" (response alternatives "Fair" and "Poor").

The overall response rate of the survey was 54% (*n *= 7238). More females (59%) than males (49%) responded to the survey. The highest response rate (69%) was from respondents in the oldest age group (65-84 years). The youngest age group (18-29 years) had the lowest response rate (40%).

All statistical analyses were adjusted for survey design and non-responses [19]. The final weights that were used in the analyses were calibrated at Statistics Sweden according to respondents' country of birth, civil status, level of education and occupation. To determine categorical differences between groups (i.e. sexes, age groups etc.), Pearson's chi-squared test was used. A *p*-value below 0.05 was regarded as significant. All statistical analyses were performed by SPSS version 15.0.

## Results

About 25% of the adult population in Östergötland was categorised as physically active (Table 1). Higher activity levels were associated with younger age groups, higher education levels, higher income levels, better self-reported general health status, and lower body mass index (BMI).

**Table 1:** Physical activity levels in the adult population (18-84) years of Östergötland in 2006

	Physical activity level					
	
	*n*	Low active (%)	Somewhat active (%)	Moderately active (%)	Physically active (%)	*p*-value
Total	6966	11	27	38	25	
Sex						< 0.01
Females	3802	10	25	40	24	
Males	3164	11	29	35	25	
Age groups (years)						< 0.01
18-29	1294	9	16	34	41	
30-44	1589	10	27	36	27	
45-64	1948	10	31	39	21	
65-84	2135	14	33	40	12	
Education						< 0.01
Compulsory school	2059	16	34	38	13	
Upper secondary school	3293	10	26	37	27	
Post-secondary school	1446	6	21	38	35	
Self-reported economy						< 0.01
Good	3865	9	26	39	27	
Neither good nor poor	2042	12	28	36	24	
Poor	908	15	30	36	19	
General health						< 0.01
Good	5456	8	24	39	29	
Poor	1456	20	36	33	11	
Body mass index (BMI) (kg/m^2^)						< 0.01
<25	3400	8	22	38	32	
25.0-29.9	2373	11	32	38	20	
>30	877	20	36	33	11	

Intentions to change varied among respondents. Table 2 shows that 37% of the respondents had no intention of changing their physical activity level, 36% had thought about change, but "not just now", and 27% were determined to change "right now". Lower intention to change was mainly associated with increased age and lower education levels.

**Table 2:** Intention to change physical activity level in the adult population (18-84 years) of Östergötland in 2006

	Have you considered increasing your physical activity?				
	
	n	"No, I have no intention to change" (%)	"Yes, I have thought about change but not just now" (%)	"Yes, I am determined to change right now" (%)	p-value
Total	6569	37	36	27	
Sex					< 0.01
Females	3552	35	36	29	
Males	3017	39	37	24	
Age groups (years)					< 0.01
18-29	1289	24	38	38	
30-44	1580	24	41	34	
45-64	1883	41	37	21	
65-84	1817	64	25	11	
Education					< 0.01
Compulsory school	1782	50	33	17	
Upper secondary school	3215	34	38	29	
Post-secondary school	1422	32	37	31	
Self-reported economy					< 0.01
Good	3667	40	34	26	
Neither good nor poor	1908	36	39	25	
Poor	861	30	40	31	
General health					< 0.01
Good	5243	36	36	28	
Poor	1284	40	40	21	
Body mass index (BMI) (kg/m^2^)					< 0.01
<25	3270	42	34	24	
25-29.9	2229	34	38	28	
>30	796	23	44	33	
Activity level					< 0.01
Low active	625	27	54	19	
Somewhat active	1699	26	47	27	
Moderately active	2530	39	34	27	
Physically active	1543	49	22	29	

Respondents ranked health-related behaviours they considered to be most important to change "right now". Physical activity was the leading item on this list, with 28% of all participants choosing this item. There were however differences between the groups; with results ranging from 12% up to 41% (see Table 3). Moreover, 24% considered "Lose weight" to be the most important health behaviour change "right now", 16% answered "Healthier eating", 5% "Quit smoking", 2% "Lowering alcohol consumption" and 1% "Quit taking snuff". "Tobacco use", presented here as smoking and use of snuff, was considered to be the most important health-related behaviour to change by 33% of the smokers, and by 9% of the snuff users.

**Table 3:** Priorities in the adult population (18-84 years) regarding the need to increase physical activity levels, the need for external support to increase physical activity levels, and the anticipated venues for this support

	Most important to change			Support to change			Support setting			
	
	n	Physical activity (%)	p-value	n	Want support (%)	p-value	n	Health care* (%)	Other** (%)	p-value
Total	6862	28		1727	15		244	50	50	
Sex			< 0.01			< 0.01				0.46
Females	3743	26		925	20		154	52	48	
Males	3119	30		802	11		90	47	53	
Age groups (years)			< 0.01			0.08				< 0.01
18-29	1285	40		476	14		63	19	81	
30-44	1574	38		578	13		87	47	53	
45-64	1929	21		419	18		62	71	29	
65-84	2074	12		254	19		32	77	23	
Education			< 0.01			0.60				0.21
Compulsory school	2003	14		285	13		32	69	31	
Upper secondary school	3276	29		901	14		125	46	54	
Post-secondary school	1406	41		514	16		80	53	48	
Self-reported economy			< 0.01			< 0.01				0.09
Good	3804	29		980	13		117	59	41	
Neither good nor poor	1998	26		472	14		71	43	57	
Poor	899	27		243	25		51	38	62	
General health			< 0.01			< 0.01				0.08
Good	5381	30		1466	13		174	46	54	
Poor	1429	18		253	30		70	63	37	
Body mass index (BMI) (kg/m^2^)			< 0.01			< 0.01				0.69
<25	3298	36		1033	13		125	48	52	
25-29.9	2365	24		525	15		73	51	49	
>30	880	15		111	27		34	58	42	
Activity level			< 0.01			< 0.01				< 0.01
Low active	690	31		205	23		49	64	36	
Somewhat active	1792	33		552	17		89	58	42	
Moderately active	2612	26		627	14		71	47	53	
Physically active	1558	24		317	10		32	20	80	

* including; primary health care, hospital, occupational health services and pharmacy

** including Internet and free text options not related to the health care service.

Among those ranking physical activity as the most important behaviour to change, 15% answered that they wanted or needed support to make this change (see Table 3). Factors associated with wanting or needing support included female sex, lower physical activity, obesity, poor health, and lower self-reported income levels. Half of the respondents, when identifying primary sources of support for change, listed assorted health care providers. The highest percentage listed support from PHC providers as the primary source of support for change. These responses were associated with older age (especially females) and those describing themselves as inactive.

Almost all (97%) participants agreed that it is the individual's own responsibility to ensure a sufficient physical activity level. However, three out of four (76%) also thought that health care providers had a great responsibility to promote physical activity levels among patients. Such responses were associated with higher education levels, higher income levels, better general health, and higher activity levels amongst respondents. About half of respondents (47%) also thought that health care providers had high responsibility to promote physical activity in the general population. Factors associated with agreement on this statement included older age, higher income levels, and poor general health.

## Discussion

The present study is one of only a few Swedish large scale population surveys on lifestyle related issues. We found that one-quarter of the population was categorised as physically active. This is consistent with findings from two previous studies that include Swedish populations: the Eurobarometer study from 2002, and a population survey in the same region as in the present study from 1999. Both studies report that 23% of the respondents were categorised as physically active [20,21].

Results from the survey's question about intention to change the level of physical activity showed that approximately one third of the population had no intention to change; one third had thought about change and one third were determined to change their physical activity level. This distribution gives a good overview about the willingness to change the level of physical activity in the population. Information about differences within sub-groups can be very useful when planning physical activity interventions. Thus, we found that two groups had minimal intentions to change their physical activity levels: those in the older age groups and those with lower education levels. This finding indicates that interventions to achieve increased physical activity in these groups require specialised approaches to enhance their motivation.

In the present study, the respondents' current activity levels were related to intention to change, but the results are somewhat ambiguous. Among respondents categorised as physically active, half answered that they had no intention to change, which indicates that they were satisfied with their current activity level. On the other hand, half of those included in the physically active group were thinking about or were determined to change their activity levels. More than half of those categorised as low active had " thought about change but not just now", with about one-fifth determined to make improvements in physical activity levels. These findings indicate that there may be subgroups in each category that are ready to take on additional activity, and that would possibly benefit from support from PHC providers. Identifying other markers that indicate strong readiness to change physical activity levels would help pinpoint which groups should be targeted.

More than one out of four respondents (28%) considered increased physical activity to be the most important health-related behaviour to change "right now", more important than losing weight, making healthier eating selections, quitting tobacco use or reducing alcohol consumption. Among respondents who considered physical activity to be the most important health-related behaviour to change, 15% also reported that they would welcome active, targeted support to increase their physical activity level. This finding does however not include those identifying other behaviours than physical activity as the most important to change "right now". Thus it may not mirror the opinion of the entire population. Still, the figures give an indication about the desired levels of support requested by different groups in the population. Of respondents who might be assumed to be in greatest need of increased activity (i.e. respondents reporting poor general health, BMI>30, and inactivity) more than one-quarter wanted support to increase their physical activity. This group also, to a larger extent, requested support from health care providers to increase their physical activity level.

Although most respondents stated that they were personally responsible for ensuring that they were physically active, a large majority among all sub-groups also felt that health care providers held responsibility for supporting physical activity both among patients and in the broader population. This finding is in line with a study by Albright et al. that showed that patients not only seek lifestyle advice from their health-care providers, but also anticipate discussions of such issues as part of their medical care [22]. Moreover, a Swedish study by Johansson et al. found that patients who receive support for health behaviour change are more satisfied with their consultation than patients who are not offered this type of information [23].

The results of the current study show that elderly and patients with poor general health, high BMI, and lower activity levels were the groups with the highest self-reported wish or need for support from health care providers. These findings are interesting from a professional to patient as well as from a public health point of view, since it is beyond dispute that it is important to increase physical activity levels in the most inactive groups in society. Even small increases in activity level among sedentary populations have greater potential to influence public health than increased activity levels of those who are already active [24,25]. It is important, both for the individual and the society, to find physical activity interventions that are effective for vulnerable groups that are at high risk of developing health behaviour-related diseases. Many physical activity interventions appear to reach primarily those in good health who are already active [26]. Appropriate interventions targeting disadvantaged groups will serve to reduce health disparities in the population rather than increase them [27].

Our findings indicate that health care-based physical activity interventions are likely to be welcomed by those patients in most need of increased physical activity. Health care providers working in primary health care settings, geographically distributed across the nation, have broad access to the adult population. They are in a natural position to share information about interventions aiming to improve the overall health status within the population, particularly among those with health problems [28,29]. In Sweden, approximately 70% of the population consult a primary health care provider each year [30]. A targeted use of interventions such as PARs can help the least active patients by outlining step-by-step approaches, which takes age, gender, current activity level, activity history, motivation, and health conditions into consideration, ensuring that the physical activity levels are appropriate,

Many different interventions are available to improve physical activity in the general population today and their results have multiple effects when they are combined. Our study focuses on identifying patients who would benefit most from support by health care providers to increase their physical activity levels. Extrapolation of the results of the present study may help to formulate policy recommendations: the study suggests that inactive patients will benefit most from health care support to increase their physical activity levels. Policy recommendations for health care providers may help them to serve as motivators of this less active patient group. Incentives to primary health care centres to allow for extra time during office visits, for example, may be well worth examining.

Our study has a number of limitations that must be considered when interpreting the results. The indices of physical activity level were derived from two questions about activity in daily life and exercise, resulting in a measurement that has not yet been validated. When this survey was planned there was no simple validated physical activity question available in Sweden, thus we used the index items used in a previous population survey. The survey as a whole used simple questions to fulfil the aim to monitor the general health in Östergötland County. Moreover, the overall response rate to this survey was 54%, with considerably lower proportions of respondents in certain groups, lowering the generalisability of the results. The separate questions in the survey allowed for non-response, which resulted in different totals for individual survey items (with responses ranging from 6966-6569). The distribution of the non-respondents probably also lower the survey's generalisability. For example, there were fewer responses from inactive respondents than from more active respondents to the questions about intention to change. Another weakness of the study is the quite small number of persons in some of the sub-samples (see Table 3), which may make it more difficult to detect statistically significant differences between different categories. Still, and despite previous mentioned shortcomings, the large overall number of respondents to the survey is a considerable strength of the study.

## Conclusions

The findings from this study suggest that there is considerable need for physical activity interventions in the general population. Adults feel great responsibility for their own physical activity levels, but also attribute responsibility for promoting increased physical activity to health care practitioners. In our study population, a significant number of low active adults thought about change or were determined to change their physical activity level. Health care providers might wish to target their efforts to this group of patients to achieve the largest public health impact.

## Competing interests

The authors declare that they have no competing interests.

## Authors' contributions

MEL, the first author, led the development of this article, oversaw data collection, worked with the study statistician to interpret findings, and organized the drafts and final version of this article. DSE, PN, KE, AS & PB were materially involved in development of this article's first, second, and final draft and interpretation of data. Have final approval rights to the submitted version of the article. LW, was as the leading statistician at the department and had the major responsibility for statistical analyses and was materially involved in development of this article's first, second, and final draft and interpretation of data. Have final approval rights to the submitted version of the article. All authors have read and approved the final manuscript.

## Pre-publication history

The pre-publication history for this paper can be accessed here:

http://www.biomedcentral.com/1471-2458/10/34/prepub

## Supplementary Material

Additional file 1 **Appendix A & B**. Variables used from the population survey and physical activity index including questions and response items. Click here for file
