# Supplementary material for: Is there a demand for physical activity interventions provided by the health care sector? Findings from a population survey
Source: BMC Public Health. 2010 Jan 25;10:34. doi: 10.1186/1471-2458-10-34 (PMC2832886; doi:10.1186/1471-2458-10-34)
Supplement: Additional file 1 — Appendix A & B. Variables used from the population survey and physical activity index including questions and response items. [file 1471-2458-10-34-S1.DOC]

# Appendix A: Variables used from the population survey in Östergötland 2006

| Variables | Questions | Response items |
| --- | --- | --- |
| Physical activity | Q1: Physical activity in daily life (walking, cycling to work, etc.) over the last 12 months? | A. None |
|  |  | B. A few times/week |
|  |  | C. Several times/week |
|  |  | D. Every day/almost every day |
| Physical activity | Q2: Exercise over the last 12 months, beyond physical activity in daily life? | A. Hardly anything |
|  |  | B. Light activity at least once a week |
|  |  | C. Moderate activity at least once a week |
|  |  | D. Vigorous activity on a regular basis |
| Intention to change. This set of questions included nutrition, physical activity, weight, alcohol consumption and tobacco use | Have you considered changing any health-related behaviour by increasing your physical activity? | 1. “No, I have no intention to change” |
|  |  | 2. “Yes, I have thought about change but not just now” |
|  |  | 3. “Yes, I am determined to change right now” |
| Most important to change | If you intend to change one health-related behaviour which is the most important to change “right now” | 1. Healthier eating |
|  |  | 2. Increase physical activity |
|  |  | 3. Lose weight |
|  |  | 4. Quit tobacco use |
|  |  | 5. Lower alcohol consumption |
|  |  | 6. I don’t want to change any of these habits |
| Support to change (based on the previous question) | Would you like support and help to make these changes? | Yes or no |
|  | If yes, which support systems or care providers would be most helpful in effecting change? | 1. Primary health care |
|  |  | 2. Hospital |
|  |  | 3. Dentist |
|  |  | 4. Occupational health service |
|  |  | 5. Pharmacy |
|  |  | 6. Internet |
|  |  | 7. Other; free text fill-in line |
| Responsibility. This set of questions included nutrition, physical activity, alcohol consumption and tobacco use | 1. How much responsibility do you think you have yourself to lead a healthy lifestyle regarding physical activity?  2. How much responsibility do you think the health care providers have when it comes to trying to promote physical activity, when patients visit the clinic?  3. How much responsibility do you think the health care providers have when it comes to trying to promote physical activity in the general population? | 1. Very much |
|  |  | 2. Somewhat |
|  |  | 3. Not much |
|  |  | 4. Very little |
|  |  | 5. Don’t know |

# Appendix B: Physical activity index used in the population survey in Östergötland 2006

Physical activity index levels are based on the following combinations of Q1 and Q2 in Appendix A (first letter in the combination responds to Q1):

1. Low active: A–A, B–A.

2. Somewhat active: C–A, A–B, B–B, C–B.

3. Moderately active: D–A, D–B, A–C, B–C, C–C.

4. Physically active: D–C, A–D, B–D, C–D, D–D.
